# Continuance intention to use mobile learning for second language acquisition based on the technology acceptance model and self-determination theory

**DOI:** 10.3389/fpsyg.2023.1185851

**Published:** 2023-06-29

**Authors:** Limin He, Chunming Li

**Affiliations:** School of Foreign Languages, Zhaoqing University, Zhaoqing, Guangdong, China

**Keywords:** continuance intention, mobile learning, the technology acceptance model, self-determination theory, second language acquisition

## Abstract

**Introduction:**

This study examines the factors that predict Chinese students’ continuance intention to use mobile learning for second language acquisition based on the technology acceptance model and self-determination theory.

**Method:**

One hundred seventy undergraduates have participated in the survey and the structural equation modeling is conducted to assess the validity of the integrated model and hypotheses.

**Results:**

The findings show that instructor support can significantly predict autonomy, competence and relatedness. Autonomy and competence are positively related to perceived ease of use and continuance intention to use mobile learning for English acquisition. Relatedness significantly correlates with perceived ease of use but is not directly related to learners’ continuance intention. The relationship between perceived ease of use and continuance intention to adopt mobile learning is positive and significant. In addition, the results show that instructor support, autonomy, relatedness, competence and perceived ease of use can predict Chinese students’ continuance intention to use m-learning for second language acquisition, with 70.5% of the total variance in continuance intention being explained by these five variables.

**Conclusion:**

These results thus empirically support the integrated model, which can be used as a theoretical framework in future studies on mobile learning in higher education contexts. Moreover, the results of this study have a number of practical implications for universities and instructors.

## 1. Introduction

Mobile learning (m-learning) has become increasingly popular among students in recent decades because mobile technologies successfully give students more convenience and flexibility than the traditional classroom, enabling them to learn whenever and wherever they want (Dahal et al., 2022; Jurayev, 2023). As a new e-learning approach based on mobile technology (Almaiah and Almulhem, 2018), m-learning has come to play a vital role in formal and informal education (Cheon et al., 2012). M-learning has also helped transform the traditional teacher-centered learning methods into a student-centered learning approach (Herring et al., 2016; Reddy et al., 2022). M-learning can support all kinds of learning at different stages. In particular, university and college students can easily adopt m-learning, as they tend to possess their own mobile learning tools (Diacopoulos and Crompton, 2020; Neffati et al., 2021). Overall, m-learning has become an essential teaching and learning tool in higher educational institutions, as it can positively influence learners’ attitudes toward learning and improve their perceptions of quick and easily accessible learning activities (O'bannon and Thomas, 2014).

A large body of literature has examined m-learning in higher education contexts. In particular, some studies have examined the use of mobile technology in enhancing language learning and cultivating educational practices. Nonetheless, comparatively little empirical research has examined the application and use of mobile assisted language learning (MALL) among learners in higher education institutions, particularly in developing countries (Hoi, 2020). In particular, the empirical studies that have examined Chinese undergraduates’ continuance intention to use m-learning for second language acquisition are few, and accordingly, in this regard, it is predicted that the study would be a pioneer and fill a gap in the field. Two questions were proposed in this study: What are the factors that predict Chinese undergraduates’ continuance intention to use m-learning for second language acquisition? What are the relationships among these factors? The results were then used to evaluate the validity of an integrated model based on self-determination theory (SDT) and the technology acceptance model (TAM). In addition to empirically validating the integrated model based on SDT and the TAM, which may be used as a theoretical framework in future education research, the findings of this study contribute to our understanding of Chinese students’ intentions to engage in m-learning. In line with this, this study provides a number of recommendations for instructors and campus management to encourage students to continue using m-learning for second language acquisition and to make the most of m-learning to enhance students’ language performance.

## 2. Literature review

### 2.1. Mobile learning

Scholars have provided various definitions of m-learning. Some scholars (Sharples, 2000; Yuen and Yuen, 2008) have defined m-learning as a specific learning approach based on the application of mobile technology. Hamidi and Chavoshi (2018) defined m-learning as the application of mobile technology to acquire knowledge, attitudes and skills anytime and anywhere, and thus argued that m-learning leads to behavioral changes. In fact, m-learning can simply be defined as a new type of e-learning applied through mobile technology (Almaiah and Almulhem, 2018). In line with this, m-learning has been perceived as an offshoot of e-learning that enables users to complete learning tasks using compact wireless devices (Kumar and Chand, 2019). Almaiah and Alismaiel (2019) described m-learning as a modern learning approach in which mobile devices are used to facilitate students’ learning activities by enabling them to obtain the learning contents more easily. Although scholars have provided different definitions of m-learning, it is widely agreed that m-learning is related to the application of mobile technology and devices. In this study, m-learning is described as the combined use of mobile devices and wireless communication networks to improve education and learning and enable students to freely access their study materials anytime and anywhere (O'bannon and Thomas, 2014). With the wide adoption of mobile devices in recent years, together with the continued enhancement of hardware and software devices, m-learning has become a popular learning approach. The widespread use of mobile devices in the teaching and learning process indicates a bright future (Chand et al., 2022).

M-learning is beneficial to both educators and students as it enables learning to occur at any time and in any place, allows educators to deliver any form of information to their students and helps students engage in learning activities in a more comfortable and flexible manner (Al-Emran et al., 2016; Jurayev, 2023), and advocates for student-centered learning (Reddy et al., 2022). These benefits stem from the unique characteristics of mobile technology in terms of portability, instant connection, and situational sensitivity (Sharples, 2000; Churchill and Churchill, 2008). Portability means that mobile devices can be easily transported to any place. Instant connection means that mobile devices can be used to obtain information at any time and in any place. Situational sensitivity means that mobile devices can collect simulated or real data. On account of these characteristics, mobile devices can be considered to support four specific types of learning. First, mobile technology promotes individualized learning, as it enables students to learn according to their own needs and abilities. Second, mobile devices facilitate situated learning, as students can use their mobile devices to create specific learning environments. Third, mobile devices promote collaborative learning, as students can use their mobile devices to interact and communicate with their peers. Finally, mobile technology facilitates informal learning, as it enables students to engage in extracurricular learning (Cheon et al., 2012) and obtain unique learning experiences without constraints of time and place. For these reasons, m-learning is preferred by many universities and learners in different countries. Some universities, such as Abilene Christian University, Stanford University and the University of Washington, have taken a lead in m-learning (Keller, 2011).

With the increasing use of m-learning in various disciplines, many scholars have studied the use of mobile technology in various curriculum learning activities (e.g., Ogata et al., 2009; Chiou et al., 2010; Hwang et al., 2011). In recent years, mobile technology has been extensively applied in language learning (Lai et al., 2022). In the context of second language learning, students can take advantage of the various attributes of m-learning, such as the ability to access language learning materials anytime and anywhere (Hsu and Lin, 2021), to shift language learning across several mobile platforms and learning tools, to accommodate individual student’s language learning habits (Hoi, 2020), to enhance collaborative language learning (Kukulska-Hulme and Viberg, 2018), to achieve language learning outside the classroom (Wu, 2016), to realize language learning through various engagement modes (Reinders and Pegrum, 2017), to promote learners’ interest and performance in language learning (Lai et al., 2022) and to inspire students’ profound and holistic learning experiences (Herring et al., 2016; Wang et al., 2021).

It is critically important to understand the different factors that influence the application and use of mobile technology for technology acceptance research (Teo et al., 2019). In line with this, scholars have examined the factors that affect students’ acceptance intentions and actual adoption of m-learning for language acquisition. For example, Kim and Lee (2016) studied the factors influencing the mobile-assisted language acquisition of Korean students and found that perceived usefulness (PU), perceived enjoyment, perceived ease of use (PEOU) and content reliability were significantly related to students’ acceptance of m-learning. Hoi (2020) investigated the acceptance and implementation of m-learning in language acquisition among university students in Vietnam based on the unified theory of acceptance and use of technology and found that performance expectancy and attitudes were critical factors in predicting intention and the actual use of mobile technology for language learning. However, their results indicated that the facilitating conditions were not directly related to the students’ actual adoption of m-learning. Lai et al. (2022) explored students’ use of mobile technology in self-directed language learning in Chinese universities based on the integrative behavior prediction model and found that subjective norms and attitudes exerted significant effects on students’ intentions to use mobile technology, but self-efficacy was not directly related to students’ intentions. In addition, they found that students’ actual application of mobile technology was significantly affected by self-regulation of skills and intentions.

With the ongoing advancements of mobile communications technology in China, many Chinese undergraduates have begun to use m-learning for second language acquisition. Nonetheless, the research which have examined the factors predicting Chinese students’ continuance intention to engage in m-learning for their language studies remain limited, which is the concern of this study.

### 2.2. Technology acceptance model

The TAM (Davis, 1986) was proposed to examine issues associated with the application of computer technology. Based on Fishbein and Ajzen’s (1975) theory of reasoned action, the TAM has been applied in behavioral studies on the acceptance of information systems and the factors that influence computer acceptance. The theoretical model holds that PEOU directly affects PU, and that PU and PEOU are directly and positively related to technology acceptance attitudes, thus affecting individuals’ technology acceptance intentions and actual technology use. PEOU is one of the most important components of the TAM. PEOU refers to ‘the degree to which a person believes in the ease of using a particular system’ (Davis, 1989, p. 320). Thus, the TAM predicts that PEOU plays a significant role in influencing the adoption of new technologies (Kumar and Chand, 2019).

The TAM is very concise and parsimonious (Lee and Ryu, 2013) and can explain the use of various information systems and technologies in various situations (Yousafzai et al., 2007). Moreover, its reliability, validity and applicability have been confirmed by numerous studies based on a variety of samples and contexts, such as autonomous vehicles (Yuen et al., 2021), mobile banking (Purwanto et al., 2020), telemedicine (Chau and Hu, 2001; Kamal et al., 2020), construction (Park and Park, 2020), mobile libraries (Rafique et al., 2020), blogs (Ifinedo, 2017), smartphone usage (Joo and Sang, 2013) and instant messaging (Lu et al., 2009). In addition, the TAM has recently been applied in the field of education, with some scholars using it to investigate students’ acceptance and use of information technology (e.g., Hamidi and Chavoshi, 2018; Vululleh, 2018; Almaiah and Alismaiel, 2019; Li et al., 2021). In particular, the TAM has been applied in m-learning contexts. For example, Kim and Lee (2016) used the TAM to study factors potentially influencing Korean students’ MALL usage. Harchay et al. (2017) used the TAM as a theoretical foundation to examine the use of mobile-based assessments among Tunisian students who majored in computer science and used semantic web technology. Wai et al. (2018) used a mixed methods approach based on the TAM to investigate Chinese university students’ perceptions of the application of m-learning. Buabeng-Andoh (2018) used the TAM in an empirical study on nursing undergraduates’ acceptance of m-learning in Ghana. Almaiah and Alismaiel (2019) integrated the TAM with DeLone and McLean’s model and empirically investigated the factors affecting the application of an m-learning system among university students in Jordan. Overall, these studies demonstrated the robustness of the TAM for investigating the adoption of m-learning systems.

The TAM was selected as a theoretical foundation for this study for a number of reasons. Firstly, the TAM is very concise and parsimonious (Lee and Ryu, 2013). Secondly, the TAM is the most widely used and popular model for investigating the acceptance and use of information technology systems (Davis, 1989; To and Tang, 2019). Thirdly, numerous scholars have used the TAM as a theoretical framework in the context of m-learning (e.g., Al-Emran et al., 2018; Buabeng-Andoh, 2018; Almaiah and Alismaiel, 2019). Consequently, the theoretical model has proven to be satisfactory for evaluating m-learning acceptance (Kim and Lee, 2016), and the robustness of the TAM for investigating the adoption of m-learning systems is obvious. Lastly, the TAM was widely adopted in technology acceptance studies, but its application in m-learning for language acquisition in China’s higher education is still insufficient. In this vein, this research increases the TAM’s capacity for explanation in a new setting.

It is acknowledged that TAM is the most extensively used and well-liked model for examining the acceptance of information technology systems, and however, the model ignores the factors that can impact PU and PEOU (Dishaw and Strong, 1999), and its main constructs do not fully reflect the impact of user acceptance (Hamidi and Chavoshi, 2018). Nonetheless, the predictive power of the model regarding the implementation of new technology can probably be enhanced through the integration of external constructs (Davis, 1989). Moreover, the adaptability of the TAM allows a variety of external elements to be incorporated into the model for analyzing technology acceptance in particular environments, which is where its value resides (Teo et al., 2019). Thus, self-determination theory was used in this study to overcome the limitations of TAM. Specifically speaking, SDT has been extensively employed in research of motivating behavior because it focuses on the connections between the social environment (contextual support), psychological need, motivation, and outcomes. The social environment and psychological factors are critical elements that should be taken into consideration when students’ learning intention and behaviors are studied. Accordingly, SDT is adopted to study the social and psychological factors which can influence continuance intention to use m-learning for English acquisition on the part of Chinese students.

### 2.3. Self-determination theory

First proposed by Deci and Ryan (1985) and further elaborated by subsequent scholars (Noels et al., 2000; Niemiec and Ryan, 2009), SDT mainly concerns the relationships among the social environment (contextual support), psychological need, motivation, outcomes, and well-being and has been widely used in studies of motivational behavior. The core elements of SDT are three basic psychological needs and motivation (Deci and Ryan, 2000). SDT asserts that self-motivation, including intrinsic motivation and the internalization of more self-determined types of extrinsic motivation, relies on the satisfaction of three inherent psychological needs, which are natural and innate rather than identified and learnt, and are viewed as the “nutriments or conditions that are essential to an entity’s growth” (Ryan, 1995, p. 410). These three basic needs are autonomy, competence and relatedness. Autonomy refers to the desire to feel that one’s behavior is determined by one’s own will and volition (Deci and Ryan, 1985). Competence refers to one’s desire to feel that one is skillful and effective in conducting an activity or interacting with a setting (Deci and Ryan, 1985). Relatedness denotes the need to feel connected with and valued by important or significant others (Baumeister and Leary, 1995) and a sense of belonging. SDT proposes that satisfying these needs can result in better performance (Deci et al., 2001) and is crucial for the healthy development and well-being of all individuals irrespective of culture and race (Deci and Ryan, 2000). A number of empirical studies have verified the existence of the three basic psychological needs of competence, relatedness and autonomy (Deci and Ryan, 2000). For example, Sørebø et al. (2009) showed that autonomy, competence and relatedness could explain teachers’ intentions to continue to use e-learning. Similarly, Nikou and Economides (2017) found that autonomy, competence and relatedness could predict students’ intentions to use mobile-based assessments.

SDT also posits that the social environment plays an especially important role in satisfying the three psychological needs. In particular, the lack of a supportive social context can have negative effects on an individual’s performance, development and well-being. Although individuals can be intrinsically stimulated, they still need a supportive environment to maintain and strengthen their innate drive (Ryan and Deci, 2000). According to SDT, contextual support, such as instructor support, peer support and social interaction, can play a significant role in enhancing motivation. Lai (2015) argued that instructor support was closely related to students’ autonomous learning and demonstrated that teachers provided three categories of support to students in promoting technology adoption, namely, affective support, capacity support and behavior support. In addition, Hoi and Mu (2021) classified perceived teacher support as teacher orientation support and teacher behavior support. Moreover, studies have shown that students’ acceptance and adoption of MALL can be determined by their teachers’ demonstration, guidance, expectations and recommendations with respect to mobile resources, applications and practices for language learning (Lai and Zheng, 2018; García Botero et al., 2019). In this study, instructor support is expected to play an important role in influencing Chinese students’ continuance intention to use m-learning for language study. In this context, instructor support is defined as instructors’ encouragement, recommendations and guidance on useful technology resources and strategies for using these resources for language learning (Williams and Deci, 1996).

Pintrich and Schunk (2002) argued that SDT is one of the most inclusive and empirically validated theories on motivation. In fact, SDT has been successfully applied in research on a variety of subjects in different contexts, such as work performance (Zhang et al., 2016), social networking sites (Wang et al., 2016), politics (Losier et al., 2001), health care and behavior (Williams et al., 2006; Hagger and Chatzisarantis, 2009), religion (Neyrinck et al., 2005), physical education (Standage et al., 2005), music education (Evans, 2015), online learning (Sørebø et al., 2009; Chen and Jang, 2010; Hsu et al., 2019), mobile-based assessment and teaching (Nikou and Economides, 2017; Stupniskya et al., 2018), video game enjoyment (Rogers, 2017), and virtual learning environments (Hew and Kadir, 2016; Huang et al., 2019). Moreover, SDT is regarded as an appropriate theoretical framework for examining information technology usage and intention. Hence, in this study, several SDT constructs (instructor support, relatedness, autonomy and competence) were used to investigate Chinese undergraduates’ continuance intention to use m-learning for second language acquisition.

**Figure 1:**
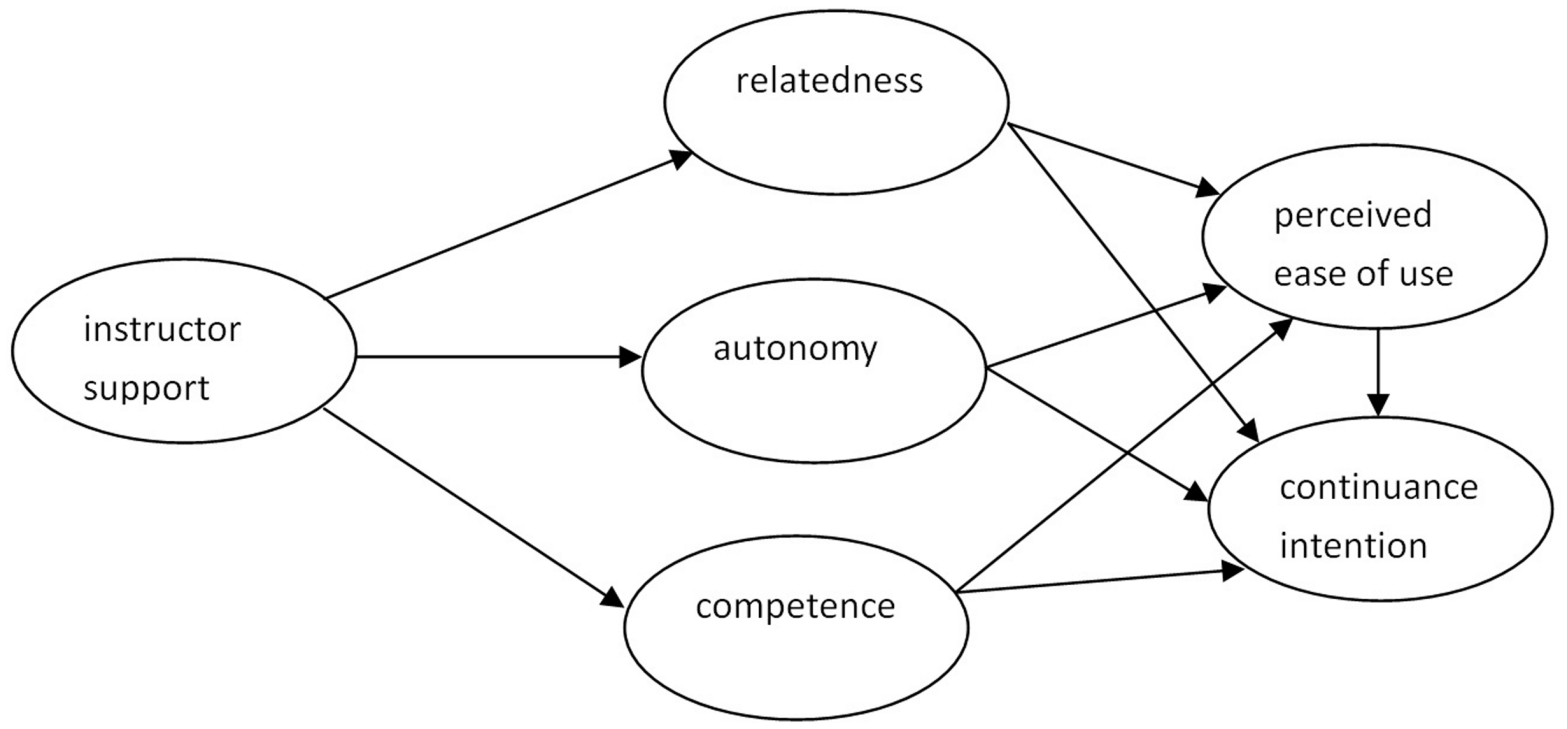
The proposed integrated model.

The following hypotheses and integrated model (Figure 1) are proposed based on the TAM and SDT.

*H1*: Instructor support is positively related to m-learning autonomy for second language acquisition.

*H2*: Instructor support is positively related to m-learning competence for second language acquisition.

*H3*: Instructor support is positively related to m-learning relatedness for second language acquisition.

*H4*: Autonomy is positively related to PEOU of m-learning for second language acquisition.

*H5*: Autonomy is positively related to learners’ continuance intention to use m-learning for second language acquisition.

*H6*: Relatedness is positively related to PEOU of m-learning for second language acquisition.

*H7*: Relatedness is positively related to learners’ continuance intention to use m-learning for second language acquisition.

*H8*: Competence is positively related to PEOU of m-learning for second language acquisition.

*H9*: Competence is positively related to learners’ continuance intention to use m-learning for second language acquisition.

*H10*: PEOU is positively related to learners’ continuance intention to use m-learning for second language acquisition.

## 3. Methodology

### 3.1. Participants and data collection

A survey was conducted by two professional surveyors who had been trained on how to conduct surveys in an advanced research methods class. The surveyors approached over 300 English majors of different grades from Zhaoqing University in Guangdong province in China, among whom 230 agreed to fill in the questionnaire. English majors were chosen to ensure that the participants clearly understood the original English version of the questionnaire. Before the participants filled in the questionnaire, the surveyors provided an objective definition of m-learning and asked the participants whether they had used m-learning for second language acquisition. The students who stated that they had never used m-learning for second language acquisition were excluded from the survey, and eventually, 182 questionnaires were collected. After excluding those with missing values, logical inconsistencies and outliers, 170 valid questionnaires were obtained for later data analysis.

The participants’ profiles were analyzed based on the frequency distributions generated by SPSS 25. Of the 170 participants, 20% were men and 80% were women. It is a common phenomenon that most English majors are women in Chinese universities. Those aged between 21 and 23 accounted for 73% of the total participants. For the question “Which m-learning tool (smartphone, laptop, iPad, kindle, others) is most frequently used to study English?”, 79.4% of the participants chose smartphone, 11.8% chose iPad and 7.1% selected laptop. These results indicate that smartphones are widely used and are the most popular technology devices among college students in China, likely on account of their convenience, availability, flexibility and especially portability. Table 1 shows the complete demographic profile of the participants.

**Table 1:** The participants’ demographic profile (number = 170).

Measure	Category	Frequency	Percent
Gender	Male	34	20
	Female	136	80
Age	18	5	2.9
	19	18	10.6
	20	23	13.5
	21	34	20.0
	22	36	21.2
	23	54	31.8
M-learning tool	Smartphone	135	79.4
	Laptop	12	7.1
	iPad	20	11.8
	Kindle	2	1.2
	Others	1	0.6

### 3.2. Measurement

This study used six constructs adopted from the literature. Six measurement items originated from Kreijns et al. (2014): Three items were adopted to measure autonomy (e.g., I have the freedom to decide when I use m-learning system in my English study), of which item1 was deleted to improve the convergent validity; Relatedness was measured by three items (e.g., I am connected to those people at school who have the same ideas about the use of m-learning system). Three items were used to measure the construct of competence (Sørebø et al., 2009; e.g., Most days I feel a sense of accomplishment from studying English with m-learning system), of which the reverse coded item3 was deleted in order to improve the convergent validity of the scale. Four items from the Learning Climate Questionnaire (Williams and Deci, 1996) were used to measure instructor support (e.g., My teacher conveys confidence in my ability to do well in English acquisition through m-learning), of which item2 was removed to improve the fit of the measurement model. A three-item scale used in Li et al. (2021) was used to measure PEOU (e.g., Learning to operate the m-learning system is easy for me). Continuance intention to adopt m-learning for second language acquisition was measured by a three-item scale (e.g., I will continue to frequently use the m-learning system for English acquisition in the future) obtained from the mature scale applied by Li et al. (2021) with some modifications. All of the items for the constructs of autonomy, competence, relatedness and instructor support were measured on a 7-point Likert scale, ranging from 1 “strongly disagree” to 7 “strongly agree”. A 9-point Likert scale was used for the items for the PEOU and intention constructs, ranging from 1 “strongly disagree” to 9 “strongly agree”. Podsakoff et al. (2003) made a study on common method biases in behavioral research and summarized the potential sources of common method biases which included common scale formats, common scale anchors and others. Likert scales of different degrees can be used to avoid method bias. Thus, we used 7-point and 9-point Likert scales at the same time to avoid common method bias.

### 3.3. Data analysis

Confirmatory factor analysis was used to evaluate both the validity and reliability of the measurement scales and to test the fit of the measurement model. Structural equation modeling (SEM) was then used to assess the proposed hypotheses and the integrated model based on SDT and the TAM. SEM was performed using IBM SPSS Analysis of Moment Structures (Amos) 24. Rather than relying on one statistical technique, SEM combines different multivariate techniques, including regression, factor analysis, path analysis, simultaneous equation and measurement theory. It can be used for research questions that focus on both the direct and indirect impacts of some factors on other variables, specify systems of causal linkages, and involve complex and multifaceted constructs that are assessed with error. SEM is suitable for complex research questions and can simultaneously specify a series of relationships and effects among unobserved and observed constructs (MacCallum and Austin, 2000). Due to the multidimensional and complicated variables used in the novel model in this study, the conceptual map could not be adequately covered by a single metric. Additionally, SEM is appropriate for examining the causal connections between the constructs of the integrated model in this study and can lower random error in measured constructs.

## 4. Results

### 4.1. Measurement model

The reliability and validity of the measurement model were examined using SPSS Amos 24. The findings showed that this measurement model satisfied all of the standard criteria. The consistency of the instruments was first evaluated by Cronbach’s alpha. The results in Table 2 show all of the values of the scales: IS, 0.791; AUT, 0.806; COM, 0.729; REL, 0.860; PEOU, 0.860; CIT, 0.858. Cronbach’s alpha values exceeded 0.7, thus confirming the reliability of each construct (Hair et al., 2011). In addition to Cronbach’s alpha, composite reliability (CR) was assessed to test the reliability of the measurements. The CR values (Table 2) for the scales were greater than the cut-off value of 0.7 (Hair et al., 2011), thus further confirming the reliability of the instruments. The convergent validity of the measurements was evaluated by the factor loadings of each item and average variance extracted (AVE). The results show that each item loading of the construct was greater than the criterion of 0.5 (Hair et al., 2011). Table 2 shows that all AVEs exceeded the minimum value of 0.5 (Fornell and Larcker, 1981), thus meeting the satisfactory level of construct validity. Discriminant validity was also evaluated by comparing the AVE with the squared latent factor correlation of the constructs. The results in Table 2 show that the square roots of the AVE were all greater than the correlation coefficients between the variables (Fornell and Larcker, 1981), thus verifying the discriminant validity of the measurements.

**Table 2:** Results of the measurement model.

	Composite reliability	Cronbach’s alpha	Average variance extracted	IS	AUT	COM	REL	PEOU	CIT
IS	0.792	0.791	0.559	**0.748**					
AUT	0.809	0.806	0.680	0.485***	**0.825**				
COM	0.732	0.729	0.577	0.597***	0.480***	**0.760**			
REL	0.861	0.860	0.675	0.701***	0.181*	0.459***	**0.822**		
PEOU	0.863	0.860	0.677	0.648***	0.523***	0.650***	0.465***	**0.823**	
CIT	0.859	0.858	0.671	0.654***	0.647***	0.684***	0.311***	0.756***	**0.819**

IS, instructor support; CIT, continuance intention; AUT, autonomy; COM, competence; REL, relatedness; PEOU, perceived ease of use.

The diagonal elements are the square roots of average variance extracted; the off-diagonal elements are correlations between variables. ***p < 0.001, *p < 0.05.

The model generated a chi-square value of 177.376 with 91 degrees of freedom (*p* < 0.001). The value of the normed chi-square was 1.949, lower than the cut-off value of 5.0 (Hair et al., 2006). The root mean square error of approximation (RMSEA) was 0.075, below the recommended criterion of 0.08 (Hair et al., 2006). In addition, the comparative fit index (CFI), incremental fit index (IFI) and Tucker–Lewis index (TLI) all surpassed the recommended value of 0.90 (Hair et al., 2006). Thus, these results (Table 3) indicated a close fit between the data and the proposed multi-dimensional measurement model.

**Table 3:** The measurement model fit indices.

	*X* ^2^	df	*X*^2^/df	IFI	TLI	CFI	RMSEA
Measurement model	177.376	91	1.949	0.943	0.923	0.942	0.075
Suggested value			<5	≥0.90	≥0.90	≥0.90	≤0.08

### 4.2. Structural model and hypothesis testing

**Figure 2:**
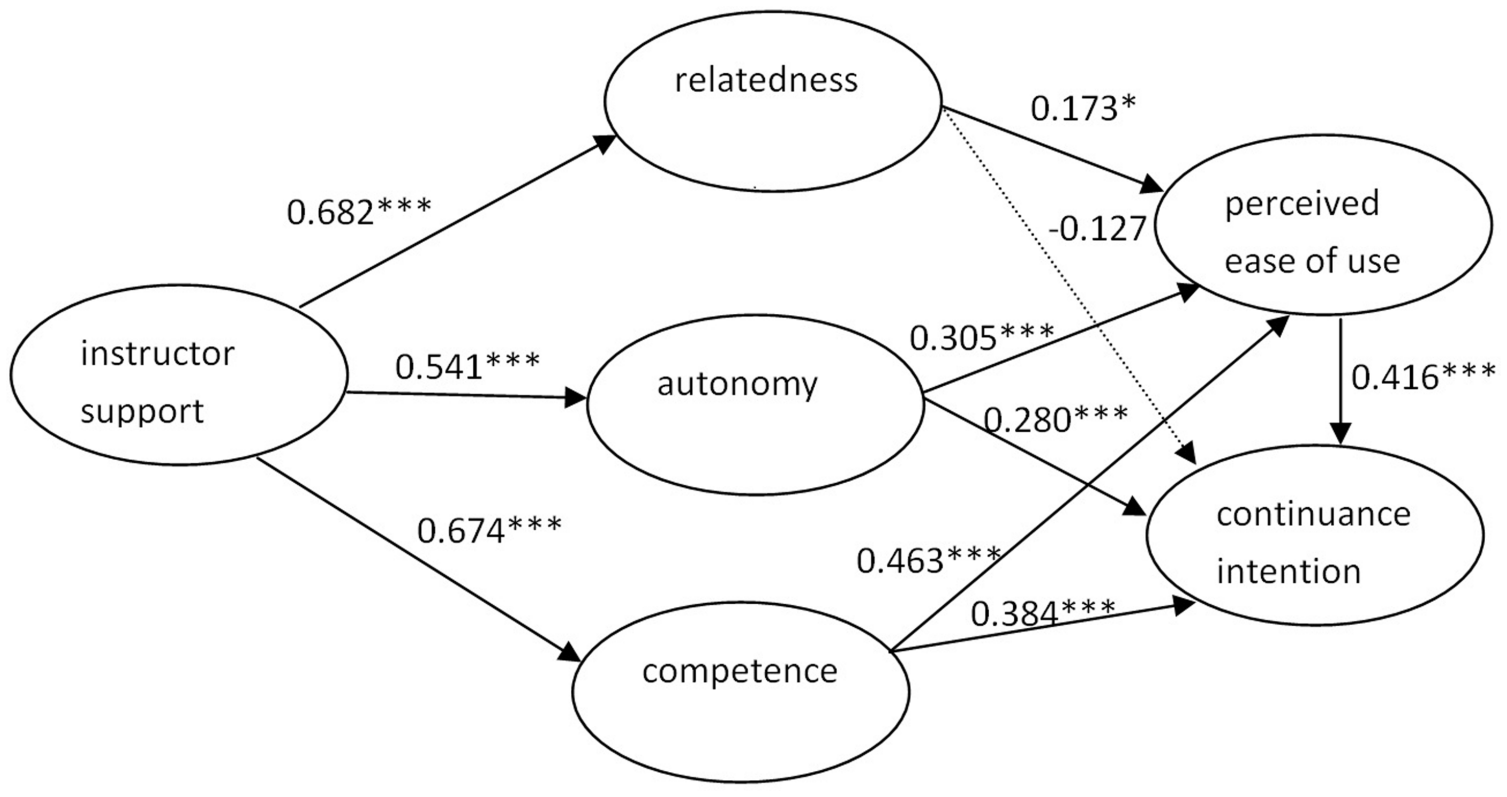
Results of SEM.

The structural model generated a chi-square value of 194.828 with 95 degrees of freedom (p < 0.001). The *X*^2^/df ratio was 2.051, below the standard value of 5.0. IFI (0.934), TLI (0.915) and CFI (0.932) surpassed the recommended criterion of 0.90. Moreover, the value of RMSEA was 0.079, below the cut-off value of 0.08. Instructor support explained 29.3% of the variance in autonomy, 45.5% of the variance in competence and 46.6% of the variance in relatedness. Instructor support, autonomy, competence and relatedness explained 55.3% of the variable PEOU. Moreover, instructor support, autonomy, competence, relatedness and PEOU explained 70.5% of the total variance in Chinese university students’ continuance intention to use m-learning for second language acquisition. The results indicated that the new integrated structural model achieved a satisfactory fit to the sample data (Table 4) and confirmed the overall relationships among the six constructs (Figure 2).

**Table 4:** The structural model fit indices.

	*X* ^2^	df	*X*^2^/df	IFI	TLI	CFI	RMSEA
Structural model	194.828	95	2.051	0.934	0.915	0.932	0.079
Suggested value			<5	≥0.90	≥0.90	≥0.90	≤0.08

Regarding the hypotheses, the results showed that except H7, all the other hypotheses were supported (Table 5). Instructor support was significantly related to autonomy (β_IS → AUT_ = 0.541, *t* = 5.353, *p* < 0.001), competence (β_IS → COM_ = 0.674, *t* = 6.919, *p* < 0.001) and relatedness (β_IS → REL_ = 0.682, *t* = 6.995, *p* < 0.001). Autonomy positively correlated with PEOU (β_AUT → PEOU_ = 0.305, *t* = 3.596, *p* < 0.001) and continuance intention to use m-learning for English acquisition (β_AUT → CIT_ = 0.280, t = 3.360, p < 0.001). Competence was significantly and positively related to PEOU (β_COM → PEOU_ = 0.463, *t* = 4.612, *p* < 0.001) and continuance intention to use m-learning for English acquisition (β_COM → CIT_ = 0.384, *t* = 3.483, *p* < 0.001). Relatedness was positively related to PEOU (β_REL → PEOU_ = 0.173, *t* = 2.022, *p* < 0.05), but not directly related to continuance intention (β_REL → CIT_ = −0.127, *t* = −1.641, *p* > 0.05). PEOU positively predicted continuance intention to use m-learning for English acquisition (β_PEOU → CIT_ = 0.416, *t* = 3.674, *p* < 0.001).

**Table 5:** The results of the hypotheses.

Hypothesis	Result
H1: Instructor support is positively related to m-learning autonomy for second language acquisition.	Supported
H2: Instructor support is positively related to m-learning competence for second language acquisition.	Supported
H3: Instructor support is positively related to m-learning relatedness for second language acquisition.	Supported
H4: Autonomy is positively related to PEOU of m-learning for second language acquisition.	Supported
H5: Autonomy is positively related to learners’ continuance intention to use m-learning for second language acquisition.	Supported
H6: Relatedness is positively related to PEOU of m-learning for second language acquisition.	Supported
H7: Relatedness is positively related to learners’ continuance intention to use m-learning for second language acquisition.	Unsupported
H8: Competence is positively related to PEOU of m-learning for second language acquisition.	Supported
H9: Competence is positively related to learners’ continuance intention to use m-learning for second language acquisition.	Supported
H10: PEOU is positively related to learners’ continuance intention to use m-learning for second language acquisition.	Supported

SEM allows not only the measurement of total and direct effects of one variable on another variable, but also indirect effects on another variable. Consequently, the results of direct and indirect effects between variables are shown in Table 6.

**Table 6:** The results of direct and indirect effects between variables.

	Autonomy	Competence	Relatedness	Perceived ease of use	Continuance intention
Instructor support	0.541***	0.674***	0.682***	(0.595***)	(0.572***)
Autonomy				0.305**	0.280* (0.127*)
Competence				0.463***	0.384** (0.193*)
Relatedness				0.173	−0.127 (0.072)
Perceived ease of use					0.416*
Continuance intention					
*R* ^2^	0.293	0.455	0.466	0.553	0.705

Values in parentheses are indirect effects.

*p < 0.05, **p < 0.01, ***p < 0.001.

## 5. Discussion

The results of this study showed that instructor support was positively and significantly related to autonomy, competence and relatedness, which is consistent with the findings of previous studies (Chen and Jang, 2010; Yu et al., 2016; Ulstad et al., 2019). For example, Yu et al. (2016) argued that teacher support improved students’ psychological needs for relatedness, competence and autonomy. Chen and Jang (2010) investigated the relationships among contextual support, need satisfaction, self-determination and course satisfaction, and empirically validated the critical importance of instructor support for need satisfaction. This indicates that when instructors encourage students to adopt m-learning, have confidence in students’ abilities to study English using m-learning and understand their students better through their own ways of using m-learning, students’ psychological needs for autonomy, relatedness and competence tend to be satisfied, which can enhance their continuance intention to implement m-learning for second language acquisition.

The findings of the current study also revealed that autonomy directly and significantly correlated with PEOU and continuance intention to use m-learning, which concurs with the findings of other studies (Nikou and Economides, 2017; Huang et al., 2019; Racero et al., 2020). For example, Nikou and Economides (2017) examined mobile-based assessments and found that autonomy had a significant and direct impact on PEOU and, consequently, influenced intention to use. Similarly, Huang et al. (2019) investigated students’ experience and motivation in a virtual learning setting and found that autonomy was strongly and positively related to behavioral intention. This indicates that when students feel a sense of autonomy in using m-learning for English study, they will perceive m-learning as easy to adopt, and at the same time, they will be inclined to continue to use m-learning for second language acquisition. Moreover, the results of the current study found that competence was positively and directly related to PEOU and continuance intention, which aligns with the findings of other studies (Nikou and Economides, 2017; Khan et al., 2018). This indicates that when students perceive themselves as competent in using m-learning for English study, they feel that m-learning is easy to adopt and are likely to continue to use m-learning in their English acquisition.

The results also showed that relatedness positively predicted PEOU, which is consistent with the prior findings (Nikou and Economides, 2017; Racero et al., 2020). This indicates that if students feel connected with important others (teachers, classmates, friends), they will perceive m-learning as easy to use for studying English. However, the findings showed that relatedness did not directly correlate with students’ continuance intention to adopt m-learning for English studies, which is contradictory with the result of the previous study (Huang et al., 2019). A possible explanation for this unexpected result might be that when deciding whether they will intend to continue using m-learning for English studies or not depends on students’ own past using experience rather than their feelings connected with others. Relatedness may play an important role in the intention of the students who have never used m-learning for English studies before. More empirical evidence is needed on this point. According to the results, PEOU directly and positively correlated with learners’ continuance intention to use m-learning, which lends credence to similar findings in the literature (Venkatesh et al., 2003; Racero et al., 2020; Li et al., 2021; Alturki and Aldraiweesh, 2022; Chen and Zhao, 2022; Rosli and Saleh, 2022). This suggests that when students perceive an m-learning system to be easy to use, they are likely to implement it for second language learning.

## 6. Conclusion

The findings show that instructor support significantly predicted autonomy, competence and relatedness. Autonomy and competence were positively related to perceived ease of use and continuance intention to use mobile learning for English acquisition. Relatedness significantly correlated with perceived ease of use but was not directly related to learners’ continuance intention. The relationship between perceived ease of use and continuance intention to adopt mobile learning was positive and significant. In addition, the results show that instructor support, autonomy, relatedness, competence and PEOU predicted Chinese students’ continuance intention to use m-learning for second language acquisition, with 70.5% of the total variance in continuance intention being explained by these five variables. These results thus empirically support the integrated TAM and SDT model.

This study has some theoretical implications. First, this study presents the first empirical investigation of Chinese students’ continuance intention to use m-learning for second language acquisition. Thus, the results of this study contribute to our knowledge of the factors that predict students’ continuance intention to use m-learning for English acquisition and contribute to the literature in this field. In addition, this study makes a significant contribution to the literature by presenting a new integrated model based on the TAM and SDT, which can be used as a theoretical framework in future studies on m-learning in higher education contexts.

The results of this study have a number of practical implications. First, the findings suggest that the instructor plays an important role in supporting students’ innate needs and continuance intention to use m-learning for second language acquisition. Therefore, it is critical to develop effective and appropriate strategies of instructor support. Instructors should provide flexible learning tasks and options (Willems, 2005) for students to make them feel that they have the freedom to choose when and how they can use m-learning for their English studies. Furthermore, instructors should frequently communicate with their students, assist them in coping with their problems and seek to bolster their confidence by offering dynamic feedback and positive assessment, which can reduce students’ anxiety and uncertainty while enhancing their continuance intention in relation to m-learning for English acquisition. Moreover, instructors should design interactive learning activities (Kreijns et al., 2003) to foster students’ interactions with their peers and teachers to satisfy their need for relatedness. This would also help satisfy students’ needs for competence, relatedness and autonomy, and lead them to perceive that m-learning is easy to use for English acquisition. Moreover, as PEOU was found to be significantly and directly related to continuance intention in this study, it is recommended that instructors or university management provide technical support for students in resolving hardware and software problems.

In terms of limitations, as this study was based on a survey of English majors from one institution in China, the findings may have limited generalizability. Thus, future studies should use broader samples from different institutions in China to improve the level of generalization. Furthermore, the data of this study were collected within a short period of time and convenience sampling method was used. Many respondents would not leave contact information, which makes it difficult for the surveyors to follow up. If conditions are permitted, future study can strive for longitudinal SEM design to assess the direction of causality. In addition, future research should examine whether other factors such as technical and parental support should be included in the model. Finally, as this study focused solely on Chinese students in higher education, a comparative study of students in different countries may provide meaningful and valuable results.

## Data availability statement

The raw data supporting the conclusions of this article will be made available by the authors, without undue reservation.

## Ethics statement

The studies involving human participants were reviewed and approved by the Academic Ethics Committee of Zhaoqing University. Written informed consent for participation was not required for this study in accordance with the national legislation and the institutional requirements.

## Author contributions

LMH and CML: conceptualization, methodology, and writing—review and editing. LMH: investigation, validation, resources, data curation, and writing original draft. CML: software, formal analysis, writing original draft, visualization, and supervision. All authors contributed to the article and approved the submitted version.

## Conflict of interest

The authors declare that the research was conducted in the absence of any commercial or financial relationships that could be construed as a potential conflict of interest.

## Publisher’s note

All claims expressed in this article are solely those of the authors and do not necessarily represent those of their affiliated organizations, or those of the publisher, the editors and the reviewers. Any product that may be evaluated in this article, or claim that may be made by its manufacturer, is not guaranteed or endorsed by the publisher.
