# Supplementary material for: Continuance intention to use mobile learning for second language acquisition based on the technology acceptance model and self-determination theory
Source: Front Psychol. 2023 Jun 29;14:1185851. doi: 10.3389/fpsyg.2023.1185851 (PMC10344694; doi:10.3389/fpsyg.2023.1185851)
Supplement: Supplementary file 1 [file Table_1.docx]

Appendix

**Table 1**

The items of the scale

| Construct | Item no. | Item description | Source |
| --- | --- | --- | --- |
| instructor support | IS1 | My teacher conveys confidence in my ability to do well in English acquisition through m-learning. |  |
|  | IS2 | My teacher listens to how I would like to do things through m-learning. | Williams & Deci, (1996) |
|  | IS3 | My teacher encourages me to ask questions on m-learning. |  |
|  | IS4 | My teacher tries to understand how I see things before suggesting a new way to do things through m-learning. |  |
| autonomy | AUT1 | I have the freedom to decide which mobile learning tool I use when I study English. | Kreijns, et al., (2014) |
|  | AUT2 | I have the freedom to decide when I use m-learning system in my English study. |  |
|  | AUT3 | I have the freedom to decide how I use m-learning system in my English study. |  |
|  | REL1 | I am connected to those people at school who have the same ideas about the use of m-learning system. |  |
| relatedness | REL2 | I am connected to those people at school who are important to me for holding on to my ideas about the use of m-learning system. | Kreijns, et al., (2014) |
|  | REL3 | I have intimate contact with those people at school with whom I spend time discussing the use of m-learning system. |  |
| perceived ease of use | PEOU1 | Learning to operate m-learning system is easy for me. |  |
|  | PEOU2 | I find it easy to get m-learning system to do what I want it to do. | Li, et al., (2021) |
|  | PEOU3 | I find m-learning system easy to use. |  |
| continuance intention | CIT1 | I will continue to use m-learning system to do different things for English acquisition. |  |
|  | CIT2 | I will continue to frequently use m-learning system for English acquisition in the future. | Li, et al., (2021) |
|  | CIT3 | I will continue to recommend others to use m-learning system for English acquisition. |  |
